# High Post-Procedural Transvalvular Gradient or Delayed Mean Gradient Increase after Transcatheter Aortic Valve Implantation: Incidence, Prognosis and Associated Variables. The FRANCE-2 Registry

**DOI:** 10.3390/jcm10153221

**Published:** 2021-07-22

**Authors:** Romain Didier, Clément Benic, Bahaa Nasr, Florent Le Ven, Sinda Hannachi, Hélène Eltchaninoff, Edward Koifman, Patrick Donzeau-Gouge, Jean Fajadet, Pascal Leprince, Alain Leguerrier, Michel Lièvre, Alain Prat, Emmanuel Teiger, Thierry Lefevre, Thomas Cuisset, Herve Le Breton, Vincent Auffret, Bernard Iung, Martine Gilard

**Affiliations:** 1 Department of Cardiology, University Hospital of Brest, 29200 Brest, France; clement.benic@chu-brest.fr (C.B.); bahaa.nasr@chu-brest.fr (B.N.); florent.leven@chu-brest.fr (F.L.V.); sinda.hannachi@chu-brest.fr (S.H.); martine.gilard@gmail.com (M.G.); 2 Cardiology Service, Rouen-Charles-Nicolle University Hospital Center, National Institute of Health and Medical Research U644, 76000 Rouen, France; helene.eltchaninoff@chu-rouen.fr; 3 Division of Cardiology, Soroka Medical Center, Beer-Sheva P.O. Box 151, Israel; eddiekoman@gmail.com; 4 Department of Cardiology and Surgery, Institut Cardiovasculaire Paris Sud, 91300 Massy, France; pdgcoeur@hotmail.com (P.D.-G.); t.lefevre@angio-icps.com (T.L.); 5 Clinique Pasteur, 31000 Toulouse, France; fajadet@interv-cardio-toul.com; 6 Cardiothoracic Surgery Department, La Pitié-Salpétrière Hospital, 75651 Paris, France; pascal.leprince@psl.ap-hop-paris.fr; 7 Cardiology and Vascular Diseases Service, Pontchaillou University Hospital Center, 35000 Rennes, France; alain.leguerrier@chu-rennes.fr (A.L.); herve.lebreton@chu-rennes.fr (H.L.B.); vincent.auffret@chu-rennes.fr (V.A.); 8 Faculty of Medecine Laënnec, University Claude Bernard Lyon, 69000 Lyon, France; ml@upcl.univ-lyon1.fr; 9 Cardiologic Hospital, 3 bd du Pr, Leclercq, CEDEX, 59037 Lille, France; alain.prat@chru-lille.fr; 10 Department of Cardiology, Henri Mondor University Hospital, Assistance Publique-Hôpitaux de Paris, 94000 Creteil, France; emmanuel.teiger@hmn.aphp.fr; 11 Department of Cardiology, La Timone University Hospital Center, Public Assistance Hospitals of Marseille, National Institute of Health and Medical Research UMR 1062, French National Institute for Agricultural Research UMR 1260, University of Aix-Marseille, 13000 Marseille, France; thomas.cuisset@ap-hm.fr; 12 Department of Cardiology, University Hospital Department Fire and Paris-Diderot University, Public Assistance Hospitals of Paris, Bichat Hospital, 75013 Paris, France; bernard.iung@bch.aphp.fr

**Keywords:** mean gradient, structural valve degeneration, TAVI, post-procedural mean gradient

## Abstract

Mean Gradient (MG) elevation can be detected immediately after transcatheter aortic valve implantation (TAVI) or secondarily during follow-up. Comparisons and interactions between these two parameters and their impact on outcomes have not previously been investigated. This study aimed to identify incidence, influence on prognosis, and parameters associated with immediate high post-procedural mean transvalvular gradient (PPMG) and delayed mean gradient increase (6 to 12 months after TAVI, DMGI) in the FRANCE 2 (French Aortic National CoreValve and Edwards 2) registry. The registry includes all consecutive symptomatic patients with severe aortic stenosis who have undergone TAVI. Three groups were analyzed: (1) PPMG < 20 mmHg without DMGI > 10 mmHg (control); (2) PPMG < 20 mmHg with DMGI > 10 mmHg (Group 1); and (3) PPMG ≥ 20 mmHg (Group 2). From January 2010 to January 2012, 4201 consecutive patients were prospectively enrolled in the registry. Controls comprised 2078 patients. In Group 1(*n* = 131 patients), DMGI exceeded 10 mmHg in 5.6%, and was not associated with greater 4-years mortality than in controls (32.6% vs. 40.1%, *p* = 0.27). In Group 2 (*n* = 144 patients), PPMG was at least 20 mmHg in 6.1% and was associated with higher 4-year mortality (48.7% versus 40.1%, *p* = 0.005). A total of two-thirds of the patients with PPMG ≥ 20 mmHg had MG < 20 mmHg at 1 year, with mortality similar to the controls (39.2% vs. 40.1%, *p* = 0.73). Patients with PPMG > 20 mmHg 1 year post-TAVI had higher 4-years mortality than the general population of the registry, unlike patients with MG normalization.

## 1. Introduction

Transcatheter aortic valve implantation (TAVI) is now a well-established alternative to conventional surgical aortic valve replacement (SAVR) in prohibitive, high-risk, and intermediate risk patients with symptomatic aortic valve stenosis [1,2,3,4,5,6]. The extension of indications for TAVI to patients at lower risk is, however, still a matter of debate in patients younger than 75 years of age, despite the last two low-risk randomized studies showing very promising results with the Sapien 3 and Evolute valves [7,8].

In order to standardize the definitions of valve- and patient-oriented durability outcomes and to enable the objective evaluation of existing and novel TAVI prostheses and to compare efficacy versus SAVR, a consensus statement was published by the European Association of Percutaneous Cardiovascular Interventions [9]. Their recommendations to define hemodynamic structural valve deterioration are based on the transprosthetic mean gradient (MG) and aortic regurgitation (AR) severity assessed by echocardiography. 

MG elevation can be detected immediately after the procedure or secondarily during the echocardiographic follow-up. The delayed mean transvalvular gradient increase (DMGI) could be more related to valve deterioration, while the immediate post-procedural transprosthetic mean gradient (PPMG) generally represents valve under-expansion, prosthesis patient mismatch, or pressure recovery and high flow. Several studies analyzed incidence and variables associated with immediate PPMG elevation, particularly in valve-in-valve procedures [10,11]. A few also studied long-term gradient progression and the impact on prognosis [12,13]. However, to our knowledge, these two different patterns of elevated gradients after TAVI have not been compared previously.

The aim of the present study was to identify the frequency, determinants, and influence on the prognosis of immediate high PPMG with DMGI in the FRANCE 2 (French Aortic National CoreValve and Edwards 2) registry.

## 2. Materials and Methods

### 2.1. Population

The design of the FRANCE 2 registry was previously described in the 1-, 3- and 5-year follow-up reports [12,14,15]. Briefly, the registry included all consecutive symptomatic patients (New York Heart Association class >II) with severe aortic stenosis (defined as valve area ≤ 0.8 cm^2^, mean valve gradient ≥ 40 mm Hg, or peak aortic jet velocity ≥ 4.0 m/s) ineligible for SAVR on heart team evaluation due to coexisting risk features. A total of 34 centers (all 33 French centers and 1 in Monaco) prospectively enrolled all patients undergoing TAVI between January 2010 and January 2012. All patients provided written informed consent for the anonymous processing of their data, and the institutional review board of the French Ministry of Health approved the registry. All patients received either a self-expandable device (CoreValve ReValving System, Medtronic, Minneapolis, MN, USA) or a balloon-expandable device (Edwards SAPIEN or SAPIEN XT prosthesis, Edwards Lifesciences, Irvine, CA, USA). The choice of prosthesis, approach (transfemoral, transapical, or subclavian), and anesthesia (general or local) was at the operator’s discretion. Antithrombotic therapy was left to each individual patient’s team to decide 

In the present study, patients without discharge transthoracic echocardiography (TTE) or without 6 or 12 months TTE were excluded from the present analysis. A total of three groups of patients were analyzed: (1) patients with PPMG < 20 mmHg without DMGI > 10 mmHg at 6 or 12 months (control group); (2) patients with PPMG < 20 mmHg and with DMGI > 10 mmHg at 6 or 12 months (Group 1); and finally, (3) patients with PPMG ≥ 20 mmHg at discharge (Group 2). 

### 2.2. Transthoracic Echocardiography (TTE) Evaluation

TTE was performed on the same day as the follow-up visits: before hospital discharge, at 30 days, 6 months, 12 months, and then annually. Valve function was assessed in terms of mean gradient, orifice area, and presence and severity of aortic regurgitation (graded from 0 to 4, with higher grades indicating greater severity). The transprosthetic mean gradient was calculated using the modified Bernoulli formula (∆*p* = 4 × V^2^ with v: velocity through aortic valve; mean gradient was calculated by averaging the instantaneous gradients over the ejection period, using the traced velocity curve, and was done by the software directly), and bioprosthesis surface was calculated using the continuity equation (AVA = (A ^LVOT^ × VTI ^LVOT^)/VTI ^AS^; with AVA: Aortic Valve Area; A ^LVOT^: Area at left ventricular outflow tract; VTI ^LVOT^: velocity time integral of flow at left ventricular outflow tract; VTI ^AS^: velocity time integral of flow through aortic valve). We chose a mean transprosthetic gradient cut-off at a ≥20 mmHg and ≥10 mmHg change from the post-procedural echocardiography, which corresponded to at least moderate hemodynamic structural valve deterioration according to the consensus statement by the European Association of Percutaneous Cardiovascular Interventions [9]. AR and valve area were not used as a component to define groups 1 and 2.

### 2.3. Follow-Up and Data Management

According to protocol, visits recording clinical status, events, and echocardiography were planned at 1 month, 6 months, and 1, 2, 3, 4 and 5 years. Data regarding clinical status, complications, and echocardiography were recorded. All adverse events, including mortality, were defined according to VARC (Valve Academic Research Consortium) criteria and were adjudicated by an independent committee. Data were recorded on a standardized electronic case-report form and was sent over the internet to a central database (Axonal). Database quality control was performed by checking data against source documents for 10% of patients in randomly selected centers. All fields were examined for missing data or outliers, and teams were asked to complete or correct data wherever possible. Outlying data were checked and excluded if erroneous; exclusion concerned less than 1% of the data. 

### 2.4. Statistical Analysis

Continuous variables were expressed as mean ± standard deviation or median ± interquartile range according to distribution. Comparison between groups of associated variables used the Student’s t-test, ANOVA or nonparametric tests for continuous variables, and the χ^2^ test or Fisher’s exact test for categorical variables. Cox proportional univariate analysis was used to identify variables associated with high PPMG or DMGI. Variables with *p* < 0.10 were selected for multivariate analysis. *p*-values ≤ 0.05 were considered to indicate statistical significance. Kaplan–Meier survival analysis was used to analyze all-cause mortality and a logrank test was used to compare mortality between the three groups. Comparisons between groups 1 and 2 and the control group were performed using the same Cox model. In the present analysis, due to the very low number of patients remaining in the groups 1 and 2 beyond 4 years, only the 4-year follow-up data were analyzed. All analyses used SAS software, version 9.2 (SAS Institute, Cary, NC, USA).

## 3. Results

**Figure 1:**
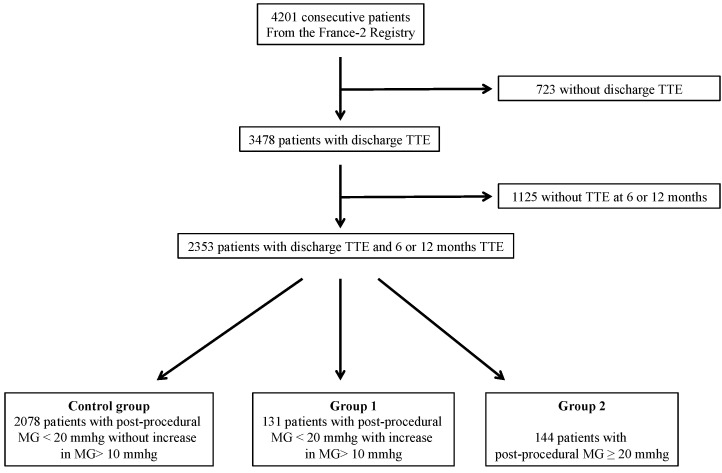
Flow chart. TTE: transthoracic echocardiography; MG: Mean Gradient.

From January 2010 to January 2012, 4201 consecutive patients underwent TAVI and were prospectively enrolled in the FRANCE 2 registry. Before hospital discharge, TTE was performed on 3478 patients. Of them, 2353 patients underwent TTE between the 6th and 12th months following intervention. The majority of patients (2209) had a PPMG < 20 mmHg at discharge. The control group consisted of 2078 patients with PPMG < 20 mmHg without DMGI > 10 mmHg at 12 months. Group 1consisted of 131 patients (5.6%) with PPMG < 20 mmHg and DMGI > 10 mmHg, and finally, Group 2 consisted of 144 patients (6.1%) with a PPMG ≥ 20 mmHg at discharge (Figure 1).

Overall clinical, procedural, and echocardiographic characteristics according to group are summarized in Table 1.

### 3.1. DMGI in Patients with PPMG < 20 mmHg (Group 1 versus Control Group) 

**Figure 2:**
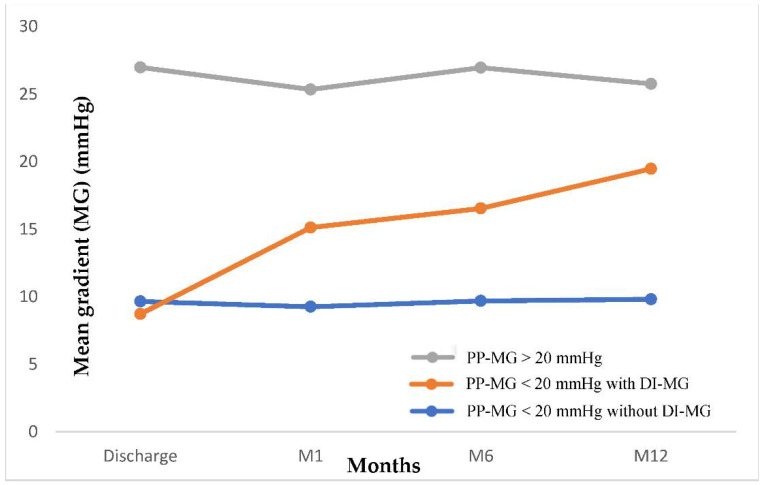
Evolution of aortic MG over time in patients with post procedural MG < 20 mmHg according to the occurrence of delayed increase in MG or not, and in patients with post procedural MG > 20 mmHg. DI-MG: Delayed increase in mean gradient; M: Month; PP-MG: Post-procedural mean gradient.

In group 1, the mean gradient increased from 8.7 ± 3.2 mmHg to 19.5 ± 8.1 mmHg during the first year of follow-up (Figure 2). A total of ninety-two patients (70.2%) had a mean gradient between 20 and 30 mmHg, 19 (14.5%) between 30 and 40 mmHg, and 1 patient had mean gradient ≥ 40 mmHg.

**Figure 3:**
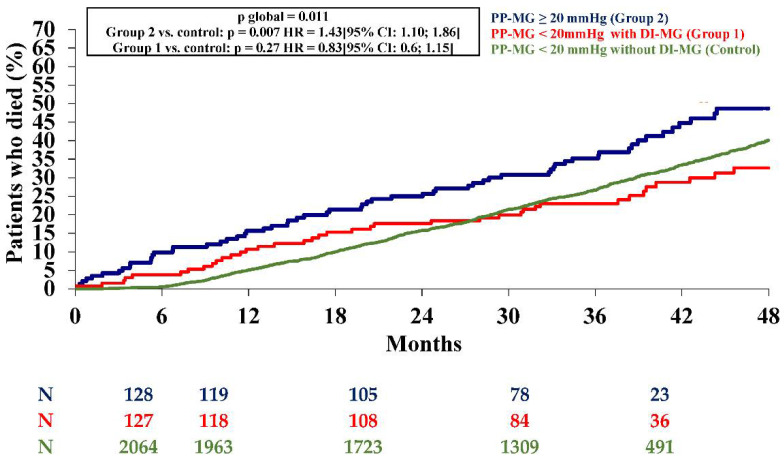
Kaplan–Meier mortality curves at 2-years from patients with post-procedural mean gradient < 20 mmHg without delayed increase in mean gradient (Control) or with delayed increase in mean gradient (Group 1) and with post-procedural mean gradient ≥ 20 mmHg (Group 2). DI-MG: delayed increase in mean gradient; M: month; PP-MG: post-procedural mean gradient.

Clinical, procedural, and echocardiographic characteristics of the patients in Group 1versus the control group are summarized in Table 1. In comparison to patients without increased mean gradient > 10 mmHg (control group), patients with DMGI had, at baseline, less severe symptoms (*p* = 0.006), higher blood pressure (*p* = 0.018), higher left ventricle ejection fraction (LVEF) (*p* = 0.032), lower mean gradient (*p*< 0.0001), and more frequent valve-in-valve procedures (*p* < 0.0001). There was no significant difference in incidence of post-procedural AR between Group 1versus the control (12.1% vs. 14.5%; *p* = 0.38). At 4 years, incidence of stroke (6.1% vs. 3.5%, *p* = 0.12) and acute heart failure (23.7% vs. 20.7%, *p* = 0.42) did not differ between Group 1 and controls. At the 4-year follow-up, there was no significant difference in all-cause mortality according to presence or absence of DMGI in patients with PPMG < 20 mmHg (*p* = 0.27 with the control group) (Figure 3).

Multivariate analysis of factors associated with DMGI is presented in Table 2. NYHA class I or II (*p* = 0.0029), absence of high blood pressure (*p* = 0.029), valve-in-valve procedures (*p* < 0.0001), valve ≤ 23 mm (*p* = 0.0019), absence of pre-procedural aspirin treatment (*p* = 0.04), and lower PPMG (*p* < 0.0001) were independently associated with the occurrence of DMGI during the first year of follow-up after TAVI.

### 3.2. Patients with PPMG ≥ 20 mmHg (Group 2 versus Control Group)

Of the patients with elevated PPMG ≥ 20 mmHg (*n* = 144: 6.1% of the total population with TTE between the 1st and 12th month following intervention), 126 underwent TTE during the following year. Echocardiographic data were missing for 18 patients (lost to follow-up or death). 

Overall, PPMG ≥ 20 mmHg was associated with higher 4-year all-cause mortality than the controls (*p* = 0.007; Figure 3). Incidences of stroke (5.6% vs. 3.8%, *p* = 0.3) and acute heart failure (24.3% vs. 20.5, *p* = 0.3) did not differ between Group 2 and the controls. In univariate analysis, patients with PPMG ≥ 20 mmHg were younger (*p* = 0.004), more often obese (*p* = 0.004) with more frequent dyslipidemia (*p* = 0.013), and a lower EuroSCORE (*p* < 0.0001) compared to the controls (Table 3). 

Pre-procedural TTE showed higher LVEF (*p* = 0.001), higher MG (*p* < 0.0001), and less frequent pre-operative AR ≥ 2 (*p* = 0.002). Smaller prostheses were more frequently used than in controls (≤23 mm; *p* = 0.001), there were more valve-in-valve procedures (*p* < 0.001), while the incidence of post-procedural AR ≥ 2 did not differ between the 2 groups (18.0 vs. 14.8; *p* = 0.3). On multivariate analysis (Table 3**)**, younger age (*p* = 0.007), BMI ≥ 35 kg/m^2^ (*p* = 0.01), dyslipidemia (*p* = 0.01), a lower EuroSCORE (*p* = 0.0007), a higher pre-procedural mean gradient (*p* = 0.0003), prosthesis size ≤ 23 mm (*p* < 0.0001) and valve-in-valve procedures (*p* < 0.0001) were associated with a high post-procedural mean gradient before discharge. 

**Figure 4:**
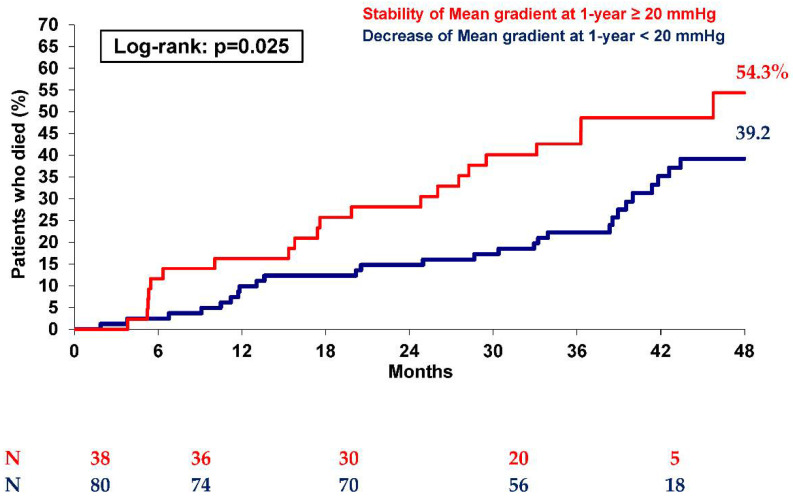
Kaplan–Meier mortality curves at 2 years of patients with post-procedural mean gradient ≥ 20 mmHg (Group 2) according to the evolution of the mean gradient at 1-year (stability or decrease).

Interestingly, at 1 year, in the initial 144 patients of Group 2, 83 showed a decrease in MG below 20 mmHg, while 43 patients still showed MG ≥ 20 mmHg. Moreover, at 4 years, as shown in Figure 4, patients with a spontaneous reduction in MG (reaching MG < 20 mmHg during the first year) had lower mortality than those who still showed a mean gradient ≥ 20 mmHg (*p* = 0.025), with mortality similar to controls (39.2% vs. 40.1%, respectively; *p* = 0.73). On the other hand, only patients still showing MG > 20 mmHg at 1 year after TAVI had higher 4-year mortality than controls (54.3% vs. 40.1%; *p* = 0.007). 

Table 4 summarizes the peri-procedural characteristics and echocardiographic findings at discharge, 6 months, and 1 year for patients with a high PPMG according to MG progression during the first year of follow-up (MG decrease to <20 mmHg or persistence of MG ≥ 20 mmHg). Notably, at discharge, the indexed aortic valve area was smaller in patients with persisting MG ≥ 20 mmHg than in patients with a decreased gradient (0.67 ± 0.2 vs. 0.90 ± 0.28; *p* < 0.001).

## 4. Discussion

The present study reports mid-term clinical and one-year echocardiographic outcomes of patients prospectively included in the FRANCE-2 registry with initial or secondary increase in PPMG, representing the largest cohort of consecutive TAVI patients with available echocardiographic follow-up. The main findings were: (1) DMGI > 10 mmHg was found after discharge at 6 or 12 months in 5.6% of patients and was not associated with higher mortality at 4 years; (2) six variables were associated with DMGI: three clinical conditions (absence of high blood pressure, NYHA class I or II, absence of pre-procedural aspirin treatment), two procedural characteristics (smaller valve size, valve-in-valve procedures), and one echocardiographic parameter (lower PPMG); (3) PPMG ≥ 20 mmHg at discharge was identified in 6.1% of the total registry population and was associated with higher all-cause mortality at 4 years (48.7% versus 40.1%; *p* = 0.005); (4) two-thirds of patients with PPMG ≥ 20 mmHg at discharge showed a less than 20 mmHg decrease at 1 year and had all-cause mortality consistent with the control population as a whole; (5) four clinical variables (younger age, BMI ≥ 35 kg/m^2^, dyslipidemia, lower EuroSCORE), two procedural variables (prosthesis size ≤ 23 mm, valve-in-valve procedure) and one echocardiographic parameter (higher pre-procedural mean gradient) were independently associated with a higher PPGM; (6) incidence of AR ≥ 2 was similar in all 3 groups.

### 4.1. DMGI in Patients with PPMG < 20 mmHg

These patients had good post-procedural TAVI results with a secondary increase in MG, possibly indicating rapid structural deterioration of the valve. In line with previous reports, this secondary increase in MG after TAVI did not seem to be associated with excess early or medium-term mortality [16,17]. Moreover, no difference was found in terms of the incidence of stroke or heart failure. Consistent with the literature, smaller valve and valve-in-valve procedures were associated with early structural valve deterioration [17,18,19]. Interestingly, in the present analysis, pre-procedural aspirin treatment was associated with lower incidence of DMGI, while clopidogrel and anticoagulation treatment were not.

### 4.2. Patients with PPMG ≥ 20 mmHg

Two-thirds of patients with PPMG ≥ 20 mmHg after TAVI (66%) recovered MG < 20 mmHg at 1 year. Only those with persistent MG > 20 mmHg at 1 year after TAVI had higher 4-year mortality than the controls (54.3% vs. 40.1%; *p* = 0.007), while patients with MG normalization had similar mortality to the controls (39.2% vs. 40.1%; *p* = 0.73). It could be argued that this sub-group of patients with a decrease of MG < 20 mmHg over the first year did not have valve under-expansion but rather transient hyperflow through the aortic valve due to left ventricular hypertrophy, which spontaneously regressed at 6 months after potential remodeling of the left ventricle. Brian et al. suggest that early regression of left ventricular hypertrophy can be observed up to 6 months after TAVI [20]. In contrast, the patients with persistent MG ≥ 20 mmHg at 1 year probably have poor valve expansion (highly calcified aortic valve) or limited expansion (more valve-in-valve procedures; 30.2% for patients with persistent MG ≥ 20 mmHg vs. 1.2% for patients with a decrease in MG < 20 mmHg, *p* < 0.001). Nevertheless, in daily practice, reaching optimal valve expansion at the end of a procedure is a major factor in reducing mortality; however, MG > 20 mmHg should not lead to systematic balloon post-dilatation since it does not necessarily correspond to the under-expansion of the valve, but possibly to transient hyperflow. Inadequate implant apposition can also lead to a mean gradient greater than 20 mmHg and can be reduced by the appropriate choice of prosthesis and the use of pre- and post-dilation to decrease the final gradient. During in-hospital ultrasound evaluation, in the case of MG > 20 mmHg, a CT-scan could be proposed to analyze valve deployment in greater depth, with repeated transthoracic ultrasound evaluation at 6 months and 1 year to assess MG progression. If under-expansion is identified on the CT-scan, additional post-dilation can be discussed to improve the mean valve gradient, which has an impact on the patient’s long-term prognosis.

### 4.3. Limitations

This study had limitations that need to be taken into consideration. First, the low number of patients remaining in Groups 1 and 2 after 4 years did not allow statistical analysis after that date. However, the FRANCE-2 population tends to have particularly high-risk baseline profiles, and the number of surviving patients falls rapidly over time. Second, the lack of systematic post-procedural CT-scans to evaluate percutaneous prosthetic valve expansion could also constitute a limitation, but this additional examination is not part of standard of care. A CT-scan could have also been interesting to search for the presence of subclinical leaflet thrombosis after implantation. In fact, the mean gradient in general is higher in patients with post-operative thrombosis and may be a risk for accelerated valve degeneration, and therefore an indication of an early gradient increase. Third, the lack of systematic core laboratory evaluation for echocardiographic assessment was another limitation, notably with no formal identification of the mechanism of transient hyperflow through the aortic valve for patients in Group 2 with an MG decrease below 20 mmHg. Finally, the present analysis includes only the earlier generation of percutaneous aortic valves, and it would be interesting to validate the findings with the latest generation of valves.

## 5. Conclusions

DMGI during the year after TAVI in patients with initial PPMG < 20 mmHg was mostly seen in small percutaneous valve and valve-in-valve procedures and was not associated with a significant increase in 4-year mortality. In contrast, patients with persistent PPMG > 20 mmHg at 1 year after TAVI had higher 4-year mortality than the control FRANCE 2 registry population in contrast to patients with MG normalization. 

## Figures and Tables

**Table 1:** Baseline, procedural, and echocardiographical characteristics.

	PP-MG < 20 mmHg without DIMG (Control) *n* = 2078	PP-MG < 20 mmHg with DIMG (Group 1) *n* = 131	PP-MG ≥ 20 mmHg (Group 2) *n* = 144	*p*-Value
Age (year)	82.6 ± 7.2	82.2 ± 6.9	80.5 ± 9.3	0.038
Male sex	1033/2078 (49.7%)	66/131 (50.4%)	69/144 (47.9%)	0.903
Body mass index (kg/m^2^)	26.21 ± 5.00	26.44 ± 4.93	26.66 ± 5.72	0.752
NYHA, III or IV	1559/2078 (75.0%)	84/131 (64.1%)	104/144 (72.2%)	
Syncope	141/2071 (6.8%)	8/131 (6.1%)	10/142 (7.0%)	0.946
Angina	347/2071 (16.8%)	14/131 (10.7%)	21/142 (14.8%)	0.167
Hypertension	1439/2071 (69.5%)	78/131 (59.9%)	113/142 (79.6%)	0.020
Diabetes	533/2071 (25.7%)	29/131 (22.1%)	29/142 (20.4%)	0.261
Dyslipidaemia	1023/2071 (49.4%)	55/131 (42%)	83/142 (58.5%)	0.023
Active smoking	67/2071 (3.2%)	1/131 (0.8%)	7/142 (4.9%)	0.126
Coronary artery disease	958/2029 (47.2%)	56/129 (43.4%)	55/138 (39.9%)	0.187
Previous CABG	386/2071 (18.6%)	26/131 (19.8%)	24/142 (16.9%)	0.816
COPB	497/2071 (24.0%)	32/131 (24.4%)	33/142 (23.2%)	0.972
Peripheral vascular disease	393/2071 (19.0%)	16/131 (12.2%)	27/142 (19.0%)	0.154
Cerebrovascular disease	205/2071 (9.9%)	13/131 (9.9%)	8/142 (5.6%)	0.248
Renal dialysis	40/2071 (1.9%)	2/131 (1.5%)	4/142 (2.8%)	0.679
Logistic EuroSCORE (%)	20.77 ± 13.28	20.48 ± 13.26	17.37 ± 11.34	0.017
STS score (%)	13.25 ± 10.98	15.52 ± 13.62	9.99 ± 9.77	<0.001
Atrial fibrillation	478/2047 (23.2%)	35/131 (26.9%)	30/144 (21%)	0.499
Permanent pacemaker	274/2075 (13.2%)	21/131 (16%)	13/143 (9.1%)	0.220
Echocardiographic findings				
LVEF (%)	53.6 ± 13.9	56.3 ± 14.8	57.0 ± 11.9	0.003
Mean AVG (mmHg)	48.57 ± 15.92	50.64 ± 17.11	55.64 ± 18.62	<0.001
Indexed AVA (cm^2^/m^2^)	0.396 ± 0.160	0.418 ± 0.219	0.380 ± 0.109	0.208
PH (sPAP > 60 mmHg)	295/1626 (18.1%)	18/95 (18.9%)	19/115 (16.5%)	0.886
Aortic regurgitation ≥ 2	363/1969 (18.4%)	29/123 (23.6%)	39/138 (28.3%)	0.009
Approach site				NA
Transfemoral	1595/2067 (77.2%)	95/130 (73.1%)	112/143 (78.3%)	
Transapical	316/2067 (15.3%)	26/130 (20.0%)	20/143 (14.0%)	
Transaortic or subclavian	119/2067 (5.8%)	6/130 (4.6%)	9/143 (6.3%)	
Type of prosthesis				
Edwards	1424/2077 (68.6%)	97/131 (74.0%)	94/143 (65.7%)	0.310
CoreValve	653/2077 (31.4%)	34/131 (26.0%)	49/143 (34.3%)	
Prosthesis size				
≤23 mm	613/2077 (29.5%)	48/131 (36.6%)	65/143 (45.5%)	<0.001
>23 mm	1464/2077 (70.5%)	83/131 (63.4%)	78/143 (54.5%)	
Previous AVR surgery	20/2071 (1.0%)	9/131 (6.9%)	15/142 (10.6%)	<0.001
Preoperative treatment				
Aspirin	1224/2071 (59.1%)	67/131 (51.1%)	83/142 (58.5%)	0.200
Clopidogrel	634/2071 (30.6%)	32/131 (24.4%)	42/144 (29.6%)	0.322
VKA	493/2071 (23.8%)	24/131 (18.3%)	29/144 (20.4%)	0.250
Discharge echocardiographic findings				
LEVF (%)	56.0 ± 12.3	57.1 ± 12.3	58.9 ± 11.6	
Mean AVG (mmHg)	10.0 ± 3.4	8.7 ± 3.2	24.7 ± 7.3	<0.001
Indexed AVA (cm^2^/m^2^)	1.054 ± 0.299	1.076 ± 0.256	0.824 ± 0.286	
Post-procedural AR ≥ 2	293/2017 (14.5%)	15/124 (12.1%)	25/139 (18.0%)	0.385

AR: aortic regurgitation; AVA: aortic valve area; AVG: Aortic valve gradient; COPB: chronic obstructive pulmonary disease; CABG: coronary artery bypass graft; DIMG: delayed increase in mean gradient; LVEF: left ventricular ejection fraction; NYHA: New York Heart Association; PP-MG: post-procedural mean gradient; PH: pulmonary hypertension; STS: Society of Thoracic Surgeons; sPAP: systolic pulmonary artery pressure; VKA: vitamin K antagonist. Values are mean ± SD or % unless otherwise specified.

**Table 2:** Variables associated with a delayed increase of mean gradient over the first year of follow-up.

	PP-MG < 20 mmHg without DIMG (Control) *n* = 2078	PP-MG < 20 mmHg with DIMG (Group 1) *n* = 131	Univariable Analysis OR [95% CI]	*p*-Value	Multivariable Analysis OR [95% CI]	*p*-Value
Age (year)	86.2 ± 7.2	82.2 ± 6.9	0.99 [0.97; 1.02]	0.552		
Male sex	49.7	50.4	0.97 [0.68; 1.39]	0.882		
Body mass index (kg/m^2^)	26.2 ± 5.0	26.4 ± 4.9	1.01 [0.98; 1.04]	0.612		
NYHA, III or IV	75.0	64.1	0.59 [0.41; 0.86]	0.006	0.56 [0.38; 0.82]	0.0029
Syncope	7.2	7.0	0.98 [0.51; 1.88]	0.946		
Angina	16.8	10.7	0.59 [0.34; 1.05]	0.072		
Hypertension	69.5	59.9	0.65 [0.45; 0.93]	0.018	0.66 [0.45; 0.96]	0.0289
Diabetes	25.7	22.1	0.82 [0.54; 1.25]	0.360		
Dyslipidaemia	49.4	42.0	0.74 [0.52; 1.06]	0.101		
Active smoking	3.2	0.8	0.23 [0.03; 1.67]	0.146		
Coronary artery disease	47.2	43.4	0.86 [0.60; 1.23]	0.402		
Logistic EuroSCORE ≥ 25	29.9	30.5	1.03 [0.70; 1.51]	0.886		
Echocardiographic findings						
LVEF (%)	53.6 ± 13.9	56.3 ± 14.8	1.02 [1.00; 1.03]	0.032		
Mean AVG (mmHg)	48.57 ± 15.92	50.64 ± 17.11	1.01 [1.00; 1.02]	0.158		
Indexed AVA (cm^2^/m^2^)	0.40 ± 0.16	0.42 ± 0.22	1.799 [0.80; 4.06]	0.158		
PH (sPAP > 60 mmHg)	18.1	18.9	1.05 [0.62; 1.79]	0.843		
Aortic regurgitation ≥ 2	18.4	23.6	1.37 [0.89; 2.10]	0.157		
Procedural characteristics						
Edwards	68.6	74.0	1.31 [0.88; 1.95]	0.190		
Prosthesis size > 23 mm	70.5	63.4	0.72 [0.50; 1.05]	0.085	0.54 [0.37; 0.80]	0.0019
Previous AVR surgery	1.0	6.9	7.57 [3.37; 16.97]	<0.001	11.40 [4.78; 27.14]	<0.0001
Preoperative treatment						
Aspirin	59.1	51.1	0.72 [0.51; 1.03]	0.074	0.67 [0.45; 0.98]	0.040
Clopidogrel	30.6	24.4	0.73 [0.49; 1.10]	0.137	0.74 [0.47; 1.14]	0.17
VKA	23.8	18.3	0.72 [0.46; 1.13]	0.153	0.65 [0.40; 1.05]	0.080
Discharge echocardiographic findings						
LEVF (%)	56.0 ± 12.3	57.1 ± 12.3	1.01 [0.99; 1.02]	0.3341		
Mean AVG (mmHg)	10.0 ± 3.4	8.7 ± 3.2	0.89 [0.84; 0.94]	<0.0001	0.87 [0.81; 0.92]	<0.0001
Indexed AVA (cm^2^/m^2^)	1.05 ± 0.90	1.08 ± 0.26	1.26 [0.60; 2.65]	0.536		
Post-procedural AR ≥ 2	14.5	12.1	0.81 [0.47; 1.41]	0.455		

AR: aortic regurgitation; AVA: aortic valve area; AVG: aortic valve gradient; COPB: chronic obstructive pulmonary disease; CABG: coronary artery bypass graft; DIMG: delayed increase in mean gradient; LVEF: left ventricular ejection fraction; NYHA: New York Heart Association; PP-MG: post-procedural mean gradient; PH: pulmonary hypertension; STS: Society of Thoracic Surgeons; sPAP: systolic pulmonary artery pressure; VKA: vitamin K antagonist. Values are mean ± SD or % unless otherwise specified.

**Table 3:** Variables associated with a post procedural mean gradient ≥ 20 mmHg.

	PP-MG < 20 mmHg *n* = 3334	PP-MG ≥ 20 mmHg *n* = 144	Univariable Analysis OR [95% CI]	*p*-Value	Multivariable Analysis OR [95% CI]	*p*-Value
Age (year)	82.9 ± 7.1	80.5 ± 9.3	0.96 [0.95; 0.98]	0.004	1.73 [1.16; 2.58]	0.007
Male sex	49.2	52.1	1.12 [0.80; 1.57]	0.497		
Body mass index (kg/m^2^)	4.9	10.5	2.29 [1.31; 4.00]	0.004	2.75 [1.17; 4.03]	0.01
NYHA, III or IV	75.1	72.7	0.89 [0.61; 1.29]	0.526		
Syncope	7.2	7.0	0.98 [0.51; 1.88]	0.946		
Angina	16.3	14.8	0.89 [0.55; 1.43]	0.624		
Hypertension	68.3	74.6	1.36 [0.93; 2.00]	0.114		
Diabetes	25.7	20.4	0.74 [0.49; 1.13]	0.162		
Dyslipidaemia	47.8	58.5	1.54 [1.09; 2.16]	0.013	1.65 [1.12; 2.44]	0.011
Active smoking	3.1	4.9	1.60 [0.73; 3.52]	0.237		
Coronary artery disease	47.0	39.9	0.75 [0.53; 1.06]	0.100		
Logistic EuroSCORE ≥ 25	31.2	18.2	0.49 [0.32; 0.76]	<0.001	0.40 [0.24; 0.68]	0.0007
Echocardiographic findings						
LVEF (%)	53.1 ± 14.2	57.0 ± 11.9	1.01 [1.01; 1.03]	0.0012		
Mean AVG (mmHg)	48.05 ± 16.11	55.64 ± 18.62	1.03 [1.01; 1.03]	<0.0001	1.03 [1.02; 1.04]	0.0003
Indexed AVA (cm^2^/m^2^)	0.398 ± 0.169	0.380 ± 0.109	0.359 [0.08; 1.73]	0.202		
PH (sPAP > 60 mmHg)	18.6	16.5	0.87 [0.53; 1.43]	0.582		
Aortic regurgitation ≥ 2	17.7	28.3	1.84 [1.25; 2.69]	0.002		
Procedural characteristics						
Edwards	66.3	65.7	0.97 [0.68; 1.39]	0.883		
Prosthesis size > 23 mm	72.0	54.5	0.47 [0.33; 0.66]	<0.001	0.43 [0.30; 0.64]	<0.0001
Previous AVR surgery	1.5	10.6	8.60 [4.67; 15.84]	<0.001	21.38 [9.94; 45.99]	<0.0001
Preoperative treatment						
Aspirin	57.7	58.5	1.07 [0.73; 1.45]	0.869	1.08 [0.72; 1.60]	0.74
Clopidogrel	29.2	29.6	1.02 [0.71; 1.47]	0.914		
VKA	23.8	20.4	0.82 [0.54; 1.25]	0.354	0.87 [0.53; 1.42]	0.57
Discharge echocardiographic findings						
LEVF (%)	55.4 ± 12.6	58.9 ± 11.6	1.02 [1.01; 1.04]	0.0019		
Mean AVG (mmHg)	9.8 ± 3.4	24.7 ± 7.3	26.70 [9.26; 76.97]	<0.0001		
Indexed AVA (cm^2^/m^2^)	1.054 ± 0.288	0.824 ± 0.286	0.024 [0.01; 0.06]	<0.0001		
Post-procedural AR ≥ 2	14.8	18.0	1.26 [0.81; 1.97]	0.302		

AR: aortic regurgitation; AVA: aortic valve area; AVG: aortic valve gradient; COPB: chronic obstructive pulmonary disease; CABG: coronary artery bypass graft; DIMG: delayed increase in mean gradient; LVEF: left ventricular ejection fraction; NYHA: New York Heart Association; PP-MG: post-procedural mean gradient; PH: pulmonary hypertension; PPM: patient-prosthesis mismatch; STS: Society of Thoracic Surgeons; sPAP: systolic pulmonary artery pressure; VKA: vitamin K antagonist. Values are mean ± SD or % unless otherwise specified.

**Table 4:** Baseline, procedural, and echocardiographic characteristics of patients with high post-procedural mean gradient (Group 2), according to the evolution of the mean gradient over the first year of follow-up (decrease < 20 mmHg or stability ≥ 20 mmHg).

	Decrease of the MG over Year < 20 mmHg *n* = 83	Stability of the MG over Year ≥ 20 mmHg *n* = 43	*p*-Value
Age (year)	80.8 ± 9.0	78.7 ± 10.4	0.260
Male sex	36/83 (43.4%)	25/43 (58.1%)	0.116
Body mass index (kg/m^2^)	26.03 ± 5.62	27.97 ± 6.09	0.042
NYHA, III or IV	56/83 (67.5%)	36/43 (83.7%)	0.051
Syncope	8/83 (9.6%)	0/43 (0%)	0.050
Hypertension	62/83 (74.7%)	32/43 (74.4%)	0.973
Diabetes	11/83 (13.3%)	15/43 (34.9%)	0.004
Dyslipidaemia	46/83 (55.4%)	29/43 (67.4%)	0.192
Active smoking	4/83 (4.8%)	3/43 (7.0%)	0.689
Coronary artery disease	32/83 (38.6%)	16/43 (37.2%)	0.883
Previous CABG	10/83 (12.0%)	14/43 (32.6%)	0.005
COPB	16/83 (19.8%)	14/43 (32.6%)	0.097
Peripheral vascular disease	18/83 (21.7%)	7/43 (16.3%)	0.470
Cerebrovascular disease	7/83 (8.4%)	0/43 (0%)	0.094
Renal dialysis	2/83 (2.4%)	1/43 (2.3%)	1
Logistic EuroSCORE (%)	15.72 ± 9.86	20.37 ± 11.97	0.033
STS score (%)	9.37 ± 8.8	9.69 ± 9.78	0.895
Atrial fibrillation	18/83 (21.7%)	7/43 (16.3%)	0.470
Permanent pacemaker	5/83 (6.0%)	6/43 (14.0%)	0.135
Echocardiographic findings			
LVEF (%)	56.1 ± 11.7	57.5 ± 11.3	0.621
Mean AVG (mmHg)	56.80 ± 19.65	54.07 ± 17.01	0.442
Indexed AVA (cm^2^/m^2^)	0.384 ± 0.107	0.370 ± 0.098	0.681
PH (sPAP > 60 mmHg)	11/67 (16.4%)	7/37 (18.9%)	0.747
Aortic regurgitation ≥ 2	25/79 (31.6%)	11/42 (26.2%)	0.532
Approach site			NA
Transfemoral	63/83 (75.9%)	36/43 (83.7%)	
Transapical	13/83 (15.7%)	4/43 (9.3%)	
Transaortic or subclavian	7/83 (8.4%)	3/43 (7.0%)	
Type of prosthesis			
Edwards	56/83 (67.5%)	26/43 (60.5%)	0.434
CoreValve	27/83 (32.5%)	17/43 (39.5%)	
Prosthesis size			
≤23 mm	39/83 (47.0%)	21/43 (48.8%)	0.844
>23 mm	44/83 (53%)	22/43 (51.2%)	
Previous AVR surgery	1/83 (1.2%)	13/43 (30.2%)	<0.001
Preoperative treatment			
Aspirin	47/83 (56.6%)	26/43 (60.5%)	0.679
Clopidogrel	23/83 (31.3%)	9/43 (20.9%)	0.217
VKA	15/83 (18.1%)	9/43 (20.9%)	0.698
Discharge echocardiographic findings			
LEVF (%)	59.2 ± 10.7	59.3 ± 12.2	0.816
Mean AVG (mmHg)	22.8 ± 3.1	27.0 ± 6.4	<0.001
Indexed AVA (cm^2^/m^2^)	0.901 ± 0.276	0.665 ± 0.216	<0.001
Post-procedural AR ≥ 2	19/81 (23.5%)	4/40 (10.0%)	0.089
6-month echocardiographic findings			
LEVF (%)	56.6 ± 13.2	62.3 ± 11.5	0.035
Mean AVG (mmHg)	12.6 ± 4.6	26.9 ± 15.1	<0.001
Indexed AVA (cm^2^/m^2^)	1.003 ± 0.297	0.923 ± 0.418	0.297
Post-procedural AR ≥ 2	15/65 (24.2%)	4/26 (16.7%)	0.569
1 year echocardiographic findings			
LEVF (%)	56.2 ± 11.2	60.9 ± 9.3	0.066
Mean AVG (mmHg)	13.0 ± 4.1	25.7 ± 9.7	<0.001
Indexed AVA (cm^2^/m^2^)	0.917 ± 0.264	0.802 ± 0.228	0.195
Post-procedural AR ≥ 2	10/62 (16.7%)	3/26 (12.0%)	0.747

AR: aortic regurgitation; AVA: aortic valve area; AVG: aortic valve gradient; COPB: chronic obstructive pulmonary disease; CABG: coronary artery bypass graft; LVEF: left ventricular ejection fraction; NYHA: New York Heart Association; PP-MG: post-procedural mean gradient; PH: pulmonary hypertension; STS: Society of Thoracic Surgeons; sPAP: systolic pulmonary artery pressure; VKA: vitamin K antagonist. Values are mean ± SD or % unless otherwise specified.

## Data Availability

The data will be not available online.
